# Development of SSR markers and genetic diversity analysis in enset (*Ensete ventricosum* (Welw.) Cheesman), an orphan food security crop from Southern Ethiopia

**DOI:** 10.1186/s12863-015-0250-8

**Published:** 2015-08-05

**Authors:** Temesgen Magule Olango, Bizuayehu Tesfaye, Mario Augusto Pagnotta, Mario Enrico Pè, Marcello Catellani

**Affiliations:** Institute of Life Sciences, Scuola Superiore Sant’Anna, Piazza Martiri della Libertà 33, 56127 Pisa, Italy; ENEA, UT BIORAD, Laboratory of Biotechnology, Research Center Casaccia, Via Anguillarese 301, 00123 Rome, Italy; Hawassa University, School of Plant and Horticulture Science, P.O.Box 5, Awassa, Ethiopia; Department of Science and Technologies for Agriculture, Forestry, Nature and Energy (DAFNE), Università degli Studi della Tuscia, Via San Camillo de Lellis, 01100 Viterbo, Italy

**Keywords:** *Ensete ventricosum*, DNA pyrosequencing, SSR markers, Genetic diversity, *Musa*, Cross-genera transferability

## Abstract

**Background:**

Enset (*Ensete ventricosum* (Welw.) Cheesman; Musaceae) is a multipurpose drought-tolerant food security crop with high conservation and improvement concern in Ethiopia, where it supplements the human calorie requirements of around 20 million people. The crop also has an enormous potential in other regions of Sub-Saharan Africa, where it is known only as a wild plant. Despite its potential, genetic and genomic studies supporting breeding programs and conservation efforts are very limited. Molecular methods would substantially improve current conventional approaches. Here we report the development of the first set of SSR markers from enset, their cross-transferability to *Musa* spp., and their application in genetic diversity, relationship and structure assessments in wild and cultivated enset germplasm.

**Results:**

SSR markers specific to *E. ventricosum* were developed through pyrosequencing of an enriched genomic library. Primer pairs were designed for 217 microsatellites with a repeat size > 20 bp from 900 candidates. Primers were validated in parallel by *in silico* and *in vitro* PCR approaches. A total of 67 primer pairs successfully amplified specific loci and 59 showed polymorphism. A subset of 34 polymorphic SSR markers were used to study 70 both wild and cultivated enset accessions. A large number of alleles were detected along with a moderate to high level of genetic diversity. AMOVA revealed that intra-population allelic variations contributed more to genetic diversity than inter-population variations. UPGMA based phylogenetic analysis and Discriminant Analysis of Principal Components show that wild enset is clearly separated from cultivated enset and is more closely related to the out-group *Musa* spp. No cluster pattern associated with the geographical regions, where this crop is grown, was observed for enset landraces. Our results reaffirm the long tradition of extensive seed-sucker exchange between enset cultivating communities in Southern Ethiopia.

**Conclusion:**

The first set of genomic SSR markers were developed in enset. A large proportion of these markers were polymorphic and some were also transferable to related species of the genus *Musa*. This study demonstrated the usefulness of the markers in assessing genetic diversity and structure in enset germplasm, and provides potentially useful information for developing conservation and breeding strategies in enset.

**Electronic supplementary material:**

The online version of this article (doi:10.1186/s12863-015-0250-8) contains supplementary material, which is available to authorized users.

## Background

Enset (*Ensete ventricosum* (Welw.) Cheesman), sometimes known as false-banana, is a herbaceous allogamous perennial crop native to Ethiopia and distributed in many parts of Sub-Saharan Africa [1–3]. Enset belongs to the genus *Ensete* of the Musaceae family. The genus *Ensete* consists of 5 or 6 species (all diploid, 2n = 2x = 18), depending on the studies [2, 3]*. E. ventricosum* is the sole cultivated member in the genus *Ensete*, and is cultivated exclusively in smallholder farming systems in southern and south-western Ethiopia [4, 5].

In Ethiopia, *E. ventricosum* is arguably the most important indigenous crop, contributing to food security and rural livelihoods for about 20 million people. Mainly produced for human food derived from starch-rich pseudostem and underground corm, the enset plant is also a nutritious source of animal fodder [6]. The crop is highly drought tolerant with a broad agro-ecological distribution and is cultivated solely with household-produced inputs [7]. Thus, enset has an immense potential for small-scale low external input and organic farming systems, particularly in the light of the climate changes. Different plant parts and processed products of several cultivated enset landraces are used to fulfil socio-cultural, ethno-medicinal and economic use-values [5–9]. Enset has an enormous potential as a food security crop that can be extended to other regions of tropical Africa, where it is known only as a wild plant [2].

Ethiopia is enset’s center of origin and holds a large number of enset germplasm collections from several geographical regions [10, 11]. There have been efforts to understand local production practices and improve the conservation and use of the genetic resources of enset in order to enhance the mostly under-exploited potential of this crop. Germplasm collection for on-farm conservation and breeding programs, mainly based on the clonal selection of landraces, have delivered considerable gains.

Despite significant progress, the genetic improvement of enset, as well as its genetic resource conservation are only based on conventional methods and have remained very slow. Primarily, complex vernacular naming systems of enset landraces by multiple ethno-linguistic communities, the nature of the vegetative propagation and the long perennial life cycle of enset make the programs laborious, time-consuming and costly [12]. Convincing evidence indicates that enset is one of the most genetically understudied food security crops with high conservation and improvement concern in Ethiopia.

The use of molecular and genomic tools is expected to substantially complement and improve ongoing conventional breeding programs and conservation efforts, by facilitating the efficient evaluation of genetic diversity, and defining the relationship and structure of the available enset germplasm stocks. DNA markers such as Inter-Simple Sequence Repeats (ISSR) [13], Random Amplified Polymorphic DNA (RAPD) [14] and Amplified Fragment Length Polymorphism (AFLP) [15] have been used to assess intra-specific genetic diversity of enset landraces. Although these markers have identified the existence of genetic diversity in enset, being dominant and difficult to reproduce, RAPD, AFLP and ISSR markers have a limited application in marker-assisted breeding, especially in heterozygous outbreeding perennial species such as enset.

Simple Sequence Repeats (SSR) are very effective DNA markers in population genetics and germplasm characterization studies due to their multi-allelic nature, high reproducibility and co-dominant inheritance [16, 17]. However, enset has historically attracted very limited research funding and has little to no genetic information available, thus the development of SSR markers has been challenging [18, 19]. To date, with the exception of reports on the cross-transferability of 11 *Musa* species SSR markers to enset [20], there are no studies on the development and application of specific enset SSRs for genetic diversity studies.

Developments in next generation sequencing (NGS) technologies provide new opportunities for generating SSR markers, especially in genetically understudied non-model crop species [19].

We report on the development of the first set of SSR markers from *E. ventricosum* using an NGS approach, on their cross-genus transferability to related taxa, and their application in assessing intra-specific genetic diversity and relationships in wild and cultivated enset accessions.

## Methods

### Plant materials and DNA isolation

Leaf tissues from 60 cultivated enset landraces and six wild individuals were collected from the enset maintenance field of Areka Agricultural Research Centre (AARC) and Hawassa University (HwU) in Ethiopia (Table 1; Additional file 1). Fresh ‘cigar leaf’ tissues, maintained in a concentrated NaCl-CTAB solution upon collection in the field, were used to isolate total genomic DNA using the GenElute™ Plant Genomic DNA Minprep Kit (Sigma-Aldrich, St. Louis, MO, USA). Cultivated enset landrace samples were originally collected from four administrative enset growing zones in southern Ethiopia: Ari, Gamo Gofa, Sidama and Wolaita. The Ari collection included five individual clones (*Entada1* to *Entada5*) of landrace *Entada*, which, unlike other enset landraces and more like banana (*Musa* spp.), produces natural suckers [21]. Wild enset is represented in our study by six individuals, *Erpha1* to *Erpha6*, all originally collected from the Dawro Zone where they are locally termed as *Erpha*. In their natural habitat, wild enset is known to propagate by botanical seeds [22].

**Table 1:** Enset and *Musa* plant materials used for marker validation, cross-transferability evaluation and genetic diversity analysis

Genus and species	Biological type/taxonomic section	Number of accessions	Geographical origin	Source
*Ensete* (*n* = 70)				
*E. ventricosum* (Welw.) Cheesman	Wild	6	Dawro, Ethiopia	AARC
*E. ventricosum* (Welw.) Cheesman	Cultivated	5	Ari, Ethiopia	HwU
*E. ventricosum* (Welw.) Cheesman	Cultivated	14	Gamo Gofa, Ethiopia	AARC
*E. ventricosum* (Welw.) Cheesman	Cultivated	5	Sidama, Ethiopia	HwU
*E. ventricosum* (Welw.) Cheesman	Cultivated	40	Wolaita, Ethiopia	AARC
*Musa* (*n* = 18)				
*M. balbisiana* Colla	Musa	4	India, Indonesia, Indonesia, NA	ITC
*M. acuminata* Colla	Musa	8	India, Malaysia, Papua New Guinea, Thailand, Philippines, Indonesia, Guadeloupe, NA	ITC
*M. ornata* Roxb.	Musa	1	NA	ITC
*M. schizocarpa* N.W. Simmonds	Musa	1	Papua new Guinea	ITC
*M. textilis* Née	Callimusa	1	NA	ITC
*Musa* cultivars		3	Papua New Guinea, India, India	ITC

*NA* Not Available, *AARC* Areka Agricultural Research Center, *HwU* Hawassa University, *ITC* International Transit Center for *Musa* collection

In addition to enset samples, 18 *Musa* accessions were also included for marker cross-transferability evaluation and as an out-group in phylogenetic analysis (Table 1; Additional file 2). The 18 *Musa* accessions represent five subspecies, including all diploid genome groups: *Musa acuminata* Colla (A genome, 2n = 22), *Musa balbisiana* Colla (B genome, 2n = 22), *Musa schizocarpa* Simmonds (S genome, 2n = 22), *Musa textilis* Nee (T genome, 2n = 20) and *Musa ornata* Robx. (2n = 22). *M. acuminata, M. balbisiana* and *M. ornata* belong to the Musa taxonomic section of the Musaceae family, whereas *M. textilis* belongs to the Callimusa section [23]. The *Musa* accessions were originally obtained from seven countries (Guadeloupe, India, Indonesia, Malaysia, Papua New Guinea, the Philippines and Thailand) and their genomic DNA samples were kindly provided by the Institute of Experimental Botany (Olomouc, Czech Republic) through a joint facilitation with Bioversity International (Montpelier, France).

### DNA sequencing and SSR detection

To identify enset-specific microsatellites, size-selected genomic DNA fragments from *E. ventricosum* landrace *Gena* were enriched for SSR content by using magnetic streptavidin beads and biotin-labeled CT and GT repeat oligonucleotides [24]. The SSR-enriched libraries were sequenced using a GS FLX titanium platform (*454* Life Science, Roche, Penzberg, Germany) at Ecogenics GmbH (Zürich-Schlieren*,* Switzerland). After trimming adapters and removing short reads (<80 bp), the generated sequences were searched for the presence of tandem simple sequence repetitive elements using in-house programs at Ecogenics. To identify long and hypervariable ‘Class I’ SSRs with a minimum motif length of 21 bp [25], SSR search parameters were set as: dinucleotide with 11 repeats, trinucleotide with 7 repeats and tetranucleotide with 6 repeats, with 100 bp maximum size of interruption allowed between two different SSRs in a sequence. The size distribution of the generated sequence reads was determined using *seqinr* package in R [26]. The generated sequence data were archived in the GenBank SRA Database [GenBank: SRR974726].

### Primer design and validation

Primer pairs flanking the identified SSRs were designed using the web interface program Primer 3 [27] by setting the following parameters: amplification product size 100 – 250 bp, and Tm difference = 1 °C. Two strategies were adopted in parallel to validate the designed primer pairs: *in silico* PCR (virtual PCR) and *in vitro* PCR amplification. All designed primer pairs were validated by the *in silico* PCR strategy using the program MFEprimer-2.0 [28]. For the PCR primer template, we referred to the less fragmented genome sequences from an uncultivated *E. ventricosum* [GenBank: AMZH01], and to the genome sequences from a cultivated *E. ventricosum* [GenBank: JTFG01] [29]. Default program settings (annealing temperature = 30–80 °C; 3’end subsequence = 9 (*k*-mer value) and product size = up to 2000 bp) were applied.

Based on the *in silico* PCR results, primer pairs were considered potentially amplifying or as a working set of primers if they i) generated a putative unique amplicon, ii) were potentially working at an annealing temperature of ≥ 50 °C, and iii) showed an absolute difference of ≤ 3 °C between the forward and its reverse. In addition, primer pairs that produced an *in silico* amplicon from the draft template genomic sequences that were different in size compared to the expected product size in our *Gena* sequence, were regarded as putatively polymorphic primers. To experimentally validate primer pairs, selected sets of primer were evaluated by *in vitro* PCR amplification using a pre-screening panel of ten enset samples. PCR was performed in a 15 μl final reaction volume containing 20 ng genomic DNA, 1X GoTaq® Reaction Buffer (manufacturer proprietary formulation containing 1.5 mM magnesium, pH 8.5 – Promega, Madison, WI, USA), 0.2 mM each of dNTPs, 0.5 U GoTaq® DNA polymerase (Promega, Madison, WI, USA), 0.4 μM of each forward and reverse primer. Reactions were performed in a Mastercycler® ep (Eppendorf, Hamburg, Germany) with the following amplification conditions: 94 °C for 5 min; 35 cycles at 94 °C for 30 s, optimal annealing temperature (Additional file 3) for 45 s and 72 °C for 45 s, and a final elongation step at 72 °C for 10 min. PCR amplification products were separated by electrophoresis in a 3 % (w/v) high resolution agarose gel in TBE buffer (89 mM Tris, 89 mM boric acid, 2 mM EDTA, pH 8.3) containing 0.5 μg/ml ethidium bromide. Electrophoresis patterns were visualized on a Gel Doc EQ™ UV-transillminator (BIO-RAD, Hercules, CA, USA) and fragment sizes were estimated using the standard size marker Hyperladder™ 100 bp (Bioline, London, England). After validation, SSR markers derived from enset genomic sequences were named with the suffix ‘Evg’ (*Ensete ventricosum* landrace *Gena*), followed by a serial number. This set of validated primers was submitted to the GenBank Probe Database, and only experimentally validated primer pairs were later used for subsequent analyses.

### SSR markers cross-genus transferability

All experimentally validated enset primer pairs were tested for cross-genus transferability on the 18 *Musa* accessions using the identical PCR setup as described earlier for enset primer pair validation. To cross-check and verify the cross-transferability of our newly developed enset markers on *Musa*, a BLAST analysis was performed using the enset sequences from which the primers were designed as queries on the whole genome sequence of banana (*Musa acuminata* ssp. malaccensis) [GenBank: CAIC01] [30]. BLAST hits were downloaded and analyzed in Clustal-W in MEGA 5.1 [31], in order to determine sequence complementarity. The informative and discriminatory ability of cross-transferred enset markers was tested by assessing the phylogenetic relationship of the 18 *Musa* accessions. A UPGMA dendrogram was constructed using Nei’s genetics distance [32] in PowerMarker 3.25 [33], and visualized with the software MEGA 5.1 [31].

### SSR genotyping

The experimentally validated enset-derived SSR markers were used to genotype the complete panel of 70 enset and 18 *Musa* accessions. Genotyping was carried out by multiplexed capillary electrophoresis using an M13-tagged forward primer (5’-CACGACGTTGTAAAACGAC-3’) at the 5’end of each primer. PCR analysis was performed with 20 ng of template genomic DNA, 1X GoTaq® Reaction Buffer (manufacturer proprietary formulation containing 1.5 mM magnesium, pH 8.5 – Promega, Madison, WI, USA), 0.2 mM each of dNTPs, 0.5 unit GoTaq® polymerase (Promega, Madison, WI, USA), 0.002 nM of M13-tailed forward primer, 0.02 nM of M13 primer labeled with either fluorescent dyes 6-Fam, Hex or Pet (Applied Biosystems®, Thermo Fisher Scientific, Waltham, MA, USA), and 0.02 nM of reverse primers in 10 μl reaction volume and amplified using a Mastercycler® ep (Eppendorf, Hamburg, Germany). The PCR amplification program consisted of an initial denaturing step of 94 °C for 3 min, followed by 35 cycles of 94 °C for 45 s, optimum annealing temperature T_*opt*_ for 1 min (Additional file 3 for optimum temperature of primers), 72 °C for 45 s, and a final extension step of 72 °C for 10 min. PCR products were diluted with an equal volume of deionized water (18 MΩcm) added to 10 μL of Hi-Di™ Formamide (Applied Biosystems®, Thermo Fisher Scientific, Waltham, MA, USA) and a 1 μL of GeneScan_500 LIZ® Size standard (Applied Biosystems®, Thermo Fisher Scientific, Waltham, MA, USA). The diluted PCR products were pooled into a multiplex set of 3 SSRs, according to their expected amplicon size and dye, and loaded onto an ABI 3730 Genetic Analyzer (Applied Biosystems®, Thermo Fisher Scientific, Waltham, MA, USA). The generated data were then analyzed using the GeneMapper® Software version 4.1 (Applied Biosystems®, Thermo Fisher Scientific, Waltham, MA, USA) and the allele size was scored in base pairs (bp) based on the relative migration of the internal size standard.

### Statistical and genetic data analyses

Observed allele frequency, polymorphic information content (PIC), observed heterozygosity (*H*_*o*_) and expected heterozygosity (*H*_*e*_) were computed by PowerMarker 3.25 [33]. The percentage of cross-genera transferability of markers was calculated at species and genus level, by determining the presence of target loci in relation to the total number of analyzed loci. Estimates of genetic differentiation (PhiPT) were computed by Analysis of Molecular Variance (AMOVA) to partition total genetic variation into within and among population subgroups using GenAlEx 6.501 [34]. To control for the correlation between observed allelic diversity and sample size of populations, rarified allelic richness (*Ar*) and private rarified allelic richness per population were estimated using rarefaction procedure implemented in the program HP-Rare 1.1 [35]. The pattern of genetic relationships among all wild enset individuals, cultivated landraces and *Musa* accessions was assessed based on the unweighted pair-group method with arithmetic mean (UPGMA) tree construction using Nei’s genetic distance coefficient [32] computed with PowerMarker 3.25 [33]. The results of UPGMA cluster analysis were visualized using MEGA 5.1 [31]. Genetic relationship and structure were further examined by a non-model-based multivariate approach, the Discriminant Analysis of Principal Components (DAPC) [36] implemented in the *adegenet* package version 1.4.1 in R [37]. We used the ‘find.clusters’ function of the DAPC to infer the optimal number of genetic clusters describing the data, by running a sequential K-means clustering algorithm for K = 2 to K = 20. After selecting the optimal number of genetic clusters associated with the lowest Bayesian Information Criterion (BIC) value, DAPC was performed retaining the optimal number of PCs (the “optimal” value following the a-score optimization procedure recommended in *adegenet*).

## Results

### Genomic sequences and SSR identification

**Fig. 1:**
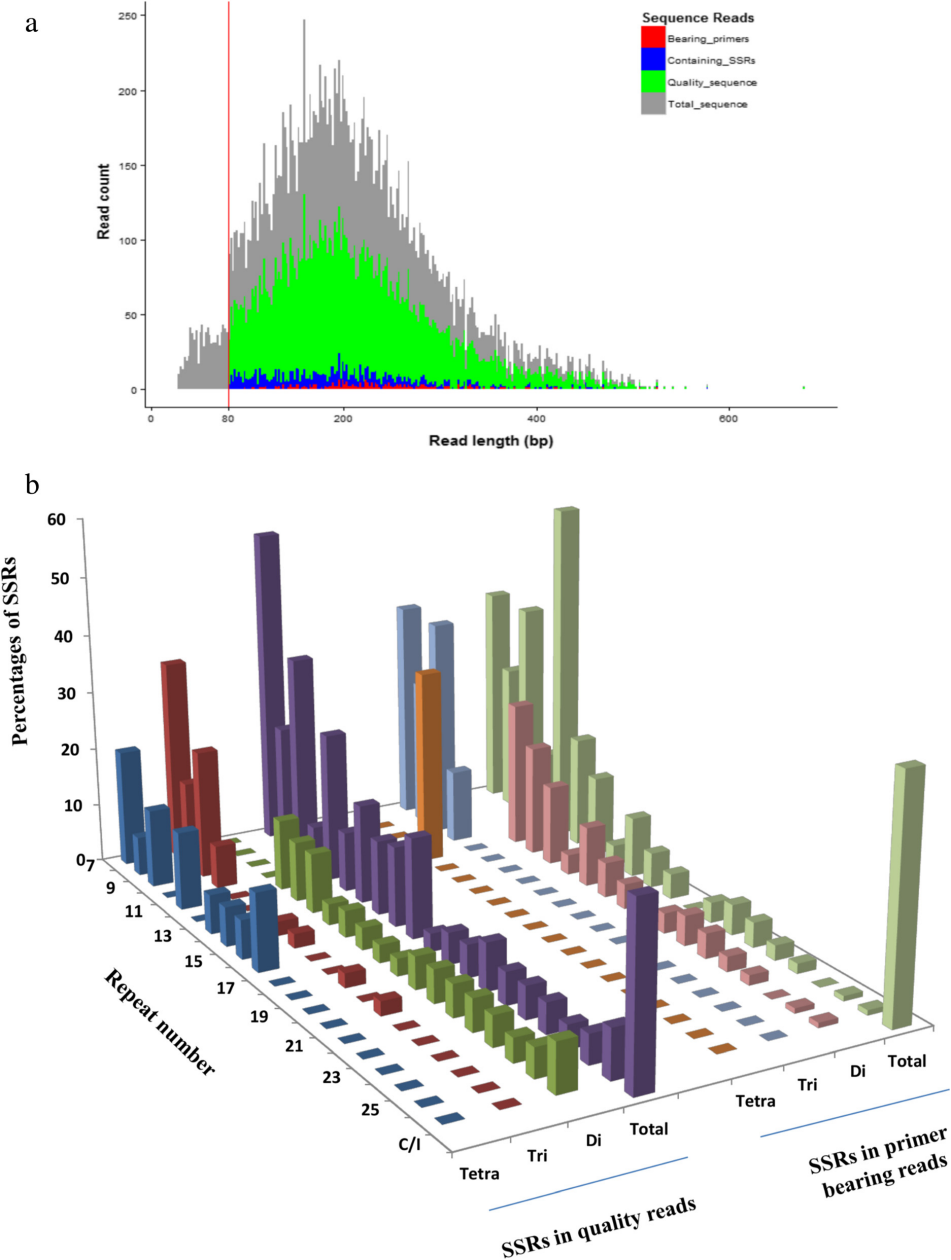
Read length distribution and SSR composition of generated sequences from enriched enset genomic libraries. **a.** Read length for overall generated reads, quality reads with minimum size of 80 bp, reads containing SSRs and bearing primer pairs, **b.** Relative frequency (%) of SSRs (di-, tri- and tetranucleotide SSRs of size > 20 bp) and number of repeats in the sequences. Repeat number with *C/I* indicates compound or interrupted SSRs

Pyrosequencing of SSR enriched *Gena* genomic libraries produced a total of 9,483 reads with lengths ranging from 29 bp to 677 bp (Fig. 1a). After trimming adaptors and removing short reads (<80 bp), a total of 8,649 non-redundant sequence reads, with an average length of 214 bp, were retained for further analysis. An automated search for only di- tri- and tetra-nucleotide SSR motifs with the desired size of > 20 bp was performed using an in-house program by Ecogenics GmbH.

This approach identified 840 reads containing a total of 900 SSRs. Two hundred and fifteen of these reads had suitable SSR flanking sequences for PCR primer design. Among these, two long reads contained two different SSRs and a sufficient stretch of flanking regions suitable for designing two different and specific primer pairs.

Overall, a total of 217 non-redundant putative SSR loci were identified from 215 reads (Additional file 3). The identified loci mainly contained SSRs with a perfect repeat structure (208 of 217 loci) and only 9 with a compound repeat structure. Perfect di-nucleotide motifs were the most abundant group, observed in 192 loci (88 %) followed by 14 tri- and 2 tetra-nucleotide motifs. The most abundant di- and tri-nucleotide motif types were (AG/GA)_n_ and (AAG/AGA/GAA)_n_ respectively, whereas (CG/GC)n, (CCG/CGG)n were the most rarely detected motifs. Figure 1b shows the distribution of SSR types, the number of repeats and their relative frequency. Table 2 summarizes the sequence data and SSR identification results.

**Table 2:** Summary of pyrosequencing data and number of identified di-, tri- and tetra- nucleotide SSR loci

Category	Numbers
Total number of reads	9,483
Total number of base-pairs	1.9 Mbp
Number of quality reads^a^	8,649
Average length quality reads	214 bp
Reads containing di- tri- and tetra-nucleotide SSR motifs with a size of > 20 bp	840
Sequence reads with SSR flanking region	215
SSR loci identified for primer-pair design	217
Perfect motif types in the identified loci	208
Dinucleotide motifs	192
Trinucleotide motifs	14
Tetranucleotide motifs	2
Compound motif types in the identified loci	9

^a^quality reads = reads with minimum size of > 80 bp

### SSR validation and marker development

To validate the 217 primer pairs, we exploited parallel *in silico* and *in vitro* PCR approaches. The *in silico* (virtual PCR) validation was carried out by scanning the partial genome sequence of an uncultivated *E. ventricosum* [GenBank: AMZH01] and the genome sequence of *E. ventricosum* landrace *Bedadit* [GenBank: JTFG01] as PCR primer template, using the program MFEprimer-2.0.

Fifty-one primers produced a potentially amplifiable product on the cultivated *Bedadit* and uncultivated enset template sequence on the basis of default parameters (see Methods). Of these, 41 primer pairs were regarded as putatively polymorphic, as they produced an *in silico* amplicon that was different in length compared to the product size observed in *Gena* sequence. Details of the *in silico* validated primer pair sequences with their SSR repeat motifs, annealing temperature, expected product size, scaffold and contig positions on template sequences are provided in Additional file 4.

Experimental *in vitro* validation was carried out by PCR on 48 randomly selected primers on a pre-screening panel of ten enset samples. Thirty-four primers produced a clear and unique amplicon, whereas 14 were discarded because of un-specific, multiple and/or unclear amplification patterns. Overall, 67 primers were validated by combining the *in silico* and the *in vitro* data, 59 of which were polymorphic. Relative to the total primer pairs tested in each of the methods, most of the primers (71 %) were validated *in vitro* compared to the *in silico* PCR (24 %).

The 67 working primer pairs were sequentially named with the suffix ‘Evg’ (*Ensete ventricosum* landrace *Gena*) followed by serial numbers and received GenBank Probe Database accession numbers from [GenBank: Pr032360175] to [GenBank: Pr032360241] (Additional file 4). Thirty-four experimentally validated SSR markers were used for further allelic polymorphism and genetic diversity analysis on the full screening panel of 70 wild individuals and enset landraces and 18 *Musa* accessions (Table 3).

**Table 3:** Characteristics of 34 polymorphic SSR markers developed in enset (Ta = annealing temperature)

Marker name	Forward primer sequence (5'–3')	Reverse primer sequence (5'–3')	Repeat motif	Size range (bp)	Ta (°C)
Evg-01	AGTCATTGTGCGCAGTTTCC	GGAGGACTCCATGTGGATGAG	(CTT)8	100–120	60
Evg-02	GGAGAAGCATTTGAAGGTTCTTG	TTCGCATTTATCCCTGGCAC	(AG)12	118–153	55
Evg-03	ACAGCATAAGCGAAATAGCAG	ACAGCATAAGCGAAATAGCAG	(AG)12	107–123	60
Evg-04	GCCATCGAGAGCTAAGGGG	GGCAAGGCCGTAAGATCAAC	(AG)21	113–147	60
Evg-05	AGTTGTCACCAATTGCACCG	CCATCCTCCACACATGCC	(GA)22	103–141	60
Evg-06	CCGAAGTGCAACACCAGAG	TCGCTTTGCTCAACATCACC	(GAA)9	202–211	60
Evg-07	GGTTGTCCTCAAGAACGTGG	TGATGCCTAATGCCTCTCCC	(GTG)9	73–94	60
Evg-08	CCATCGACGCCTTAACAGAG	TGAACCTCGGGAGTGACATAAG	(GA)21	164–190	60
Evg-09	GCCTTTCGTATGCTTGGTGG	ACGTTGTTGCCGACATTCTG	(GA)13	141–175	60
Evg-10	CAGCCTGTGCAGCTAATCAC	CAGCAGTTGCAGATCGTGTC	(AG)21	191–210	60
Evg-11	GGCCTAGTGACATGATGGTG	TGATGCTAGATTCAAAGTCAAGG	(AC)13	135–160	60
Evg-12	TGCAACCCTTTGCTGCATTC	AGCATCATTCGCCATGGTTG	(TG)14	135–154	60
Evg-13	CTTGAAAGCATTGCATGTGGC	TCACCACTGTAGACCTCAGC	(CA)14	189–229	60
Evg-14	AACCAATCTGCCTGCATGTG	GCCAGTGATTGTTGAGGTGG	(TGA)8	153–159	60
Evg-15	TCCTTTAGGTTATTTGGTTGCC	CCTTGGACATGCCTCACATC	(AG)15	110–134	55
Evg-16	GGCTAGTCCAGTTGGAAAGAG	GTAATCACCTCTGCCTTCACC	(AG)13	109–117	60
Evg-17	GCGTCTGGTATGCTCAACTG	TCGGGAATGATACAGAGGCG	(TCA)8	111–154	60
Evg-18	TCACTCCGATGGAAGGGATG	TCTCCACCATTTTAGTTGGCAC	(GAG)7	181–188	60
Evg-19	GGTATGAAAGCCACACCACC	AGTTCACCCACGCCTCAC	(GT)16	234–255	60
Evg-20	TTGCTCTCTGCTACTGACGG	CCGGTAACTTGGTGGAAGTC	(CA)17	138–148	60
Evg-21	CAGGCAACCACTGCGATATG	CAGTTGTCTCCCCAGGTGC	(CA)12	106–116	60
Evg-22	CTATCCAGGAGCCCATCTCG	ACTCTTCTCTTCGCCTGTGG	(CA)15	88–94	60
Evg-23	CCACCAAAGGGCTCCTCG	TCGGATTCTCCCGCTATTGG	(AC)13	129–143	60
Evg-24	TTTTCGGACGGTCTCTGTGG	TTCTTCTGCTGGCGTTTGAG	(TTG)8	155–162	60
Evg-25	CACGTTGATGTCGTTCCGTC	GAATCGCTTCAAGGCGTAGG	(CT)13	201–229	60
Evg-26	AAGCCATTGATGACTCCCCG	CAGTTGCACGCAGAGAAAAC	(AC)12	110–139	60
Evg-27	GCAATAGAATGGTACGGAGCG	TTTTGACTGTTCCGACGGTG	(AG)16	103–123	60
Evg-28	AAGCCACGGAATCAGCAAAC	ACCCACTACCTTTCCCTAAGC	(AC)12	201–209	60
Evg-29	GTTCGACTCGTCCAAGAAGG	ACTGTCTTAGTGATAGCCATGC	(AC)15	103–113	60
Evg-48	TAATTCTTCCCACCGGGGTC	GACCACTTACTTTTTGCACGC	(TG)12	127–133	60
Evg-49	TCCTGCACCCTCCATATTCC	TCTCTCTCTCTGATCTTCGTAGC	(GA)13	226–234	60
Evg-50	ATCTTGAACGTGGGGAAGGG	TGATACCTGGTGAGGATGCG	(TG)13	162–188	60
Evg-51	TGAATGAGTGGGGGATGCTG	AATGGATCGTTATCCAACGTG	(CAT)9	145–148	60
Evg-52	TATGGGAAGGGGATCCACAC	CAAATGCCGATAGGGACAGC	(CA)13	212–231	60

### Allelic polymorphism and genetic diversity

The 34 enset SSR markers revealed 202 alleles among the 70 wild individuals and cultivated enset landraces (Table 4). The allelic richness per locus varied widely among the markers, ranging from 2 (Evg-52) to 12 (Evg-12) alleles, with an average of 5.94 alleles. Allelic frequency data showed that rare alleles (with frequency < 0.05) comprise 43 % of all alleles, whereas intermediate alleles (with frequency 0.05–0.50) and abundant alleles (with allele frequency > 0.50) were 48 % and 9 %, respectively. Observed heterozygosity (Ho) ranged from 0.1 (Evg-24, Evg-50) to 0.96 (Evg-14), with a mean value of 0.55. Mean expected heterozygosity/gene diversity (GD) was 0.59, with a minimum of 0.10 (Evg-50) and a maximum of 0.79 (Evg-8, Evg-9). Polymorphic Information Content (PIC) values ranged from 0.09 (Evg-50) to 0.77 (Evg-8) with an average of 0.54. Allele number was positively and significantly correlated with gene diversity (GD) (r = 0.55 , *P* = 0.001) and polymorphic information content (PIC) (r = 0.64, *P* = 0.000). The association of allele number, PIC and GD with the length of SSRs (motif x number of repeats) for the 34 markers was investigated, however the correlation was not statistically significant (data not shown).

**Table 4:** Characteristics of the 34 polymorphic enset SSR markers used to assess genetic diversity in enset

Marker name	Number of alleles	Ho	GD	PIC
Evg-01	9	0.64	0.67	0.63
Evg-02	8	0.70	0.75	0.72
Evg-03	6	0.64	0.64	0.58
Evg-04	9	0.87	0.77	0.73
Evg-05	4	0.49	0.65	0.58
Evg-06	3	0.37	0.52	0.41
Evg-07	6	0.82	0.72	0.67
Evg-08	11	0.42	0.79	0.77
Evg-09	9	0.83	0.79	0.76
Evg-10	8	0.49	0.73	0.70
Evg-11	6	0.78	0.66	0.62
Evg-12	12	0.78	0.75	0.72
Evg-13	7	0.58	0.60	0.52
Evg-14	3	0.96	0.52	0.41
Evg-15	5	0.41	0.68	0.62
Evg-16	3	0.21	0.23	0.20
Evg-17	8	0.72	0.72	0.68
Evg-18	4	0.69	0.66	0.60
Evg-19	5	0.13	0.12	0.12
Evg-20	6	0.24	0.71	0.67
Evg-21	5	0.79	0.69	0.65
Evg-22	4	0.74	0.63	0.57
Evg-23	6	0.57	0.64	0.59
Evg-24	4	0.10	0.25	0.24
Evg-25	5	0.58	0.60	0.53
Evg-26	6	0.80	0.68	0.64
Evg-27	8	0.40	0.66	0.61
Evg-28	4	0.59	0.59	0.51
Evg-29	5	0.51	0.60	0.55
Evg-48	3	0.70	0.59	0.51
Evg-49	4	0.10	0.10	0.09
Evg-50	5	0.29	0.27	0.25
Evg-51	2	0.32	0.50	0.37
Evg-52	9	0.44	0.62	0.55
Mean	5.94	0.55	0.59	0.54

### Genetic relationship and structure

Genetic diversity by group, cultivated and wild enset groups as well as groups of four enset growing regions (Ari, Gamo Gofa, Sidama and Wolaita), were estimated by pooling allelic data for each population (Table 5). Polymorphic SSRs were amplified for all the 34 loci in cultivated landraces (*PPL* = 100 %), but in wild enset markers Evg-15, Evg-16 and Evg-50 amplified monomorphic SSRs (*PPL* = 91 %). Thus cultivated enset was characterized by a higher average number of alleles, *Na* and rarefied allelic richness *Ar* than wild enset. However, among the group samples of the four enset cultivating zones, rarefied allelic richness was comparable in three zones (*Ar* = 3.00 for both Gamo Gofa and Sidama, and *Ar* = 3.15 for Wolaita), with the smallest value (*Ar* = 1.62) for Ari.

**Table 5:** Diversity parameters estimated for enset population using 34 SSR markers

Diversity parameters	Cultivated and wild population			Cultivation regions				
	Cultivated (*n* = 64)	Wild (*n* = 6)	Mean ± SE	^a^Ari (*n* = 5)	Gamo Gofa (*n* = 14)	Sidama (*n* = 5)	Wolaita (*n* = 40)	Mean ± SE
Percentage of polymorphic loci (PPL%)	100	91	96 ± 4.41	59	97	88	100	86 ± 9.41
Number of different alleles (Na)	5.88	2.56	4.22 ± 0.29	1.62	3.82	3.00	4.91	3.34 ± 0.16
Rarefied allelic richness (Ar)	3.56	2.32	2.94 ± 0.44	1.62	3.00	3.00	3.15	2.69 ± 0.36
Number of effective alleles (Ne)	2.79	1.88	2.34 ± 0.11	1.59	2.41	2.52	2.64	2.29 ± 0.09
Shannon’s information index (I)	1.16	0.67	0.91 ± 0.06	0.41	0.96	0.90	1.07	0.83 ± 0.04
Observed heterozygosity (Ho)	0.55	0.55	0.55 ± 0.04	0.53	0.53	0.56	0.55	0.54 ± 0.03
Expected heterozygosity (He)	0.59	0.40	0.49 ± 0.03	0.29	0.52	0.51	0.56	0.47 ± 0.02
Private Na	3.38	0.06	1.72 ± 0.21	0.03	0.44	0.15	1.06	0.42 ± 0.10
Private Ar	1.51	0.28	0.89 ± 0.43	0.14	0.41	0.46	0.36	0.34 ± 0.07

^a^Ari population is represented by 5 individuals of the same landrace *Entada* which produces spontaneous suckers unlike other cultivated landraces

*n* = number of individuals per population

*SE* standard error

All the sample groups had at least one private allele and exhibited a similar level of observed heterozygosity. Most of the other computed diversity indices, such as the effective number of alleles per locus (*Ne*), Shannon’s information index (*I*) and expected hetrozygosity (*He*) showed a similar trend, where the Wolaita and Ari landraces showed the highest and smallest estimated value for diversity indices respectively.

AMOVA indicated that the genetic variation within groups contributed more to genetic diversity than the between groups (Table 6). In the cultivated and wild enset groups, 76 % of the total variation occurred within groups. Likewise, the proportion of variance within the growing geographic regions contributed by 84 % to the total genetic variation. The mean PhiPT value of 0.238 indicated moderate to high genetic differentiation between cultivated and wild enset groups, but a low differentiation among regions (PhiPT = 0.16). Pairwise PhiPT values for the four growing regions of cultivated enset and wild enset ranged from 0.055 (Gamo Gofa/Wolaita) to 0.644 (Wild*/*Ari) and all the PhiPT estimates were statistically significant (*P* < 0.001; data not shown).

**Table 6:** Analysis of Molecular Variance among and within populations of wild and cultivated enset as well as different growing regions

Source of variation	*df*	Sum of squares	Variance component	Percentage variation (%)	*PhiPT*
Wild and cultivated enset					
Among Pops	1	100.11	7.06	24	0.238
Within Pops	68	1537.61	22.61	76	
Growing regions					
Among Pops	3	199.15	3.91	16	0.16
Within Pops	60	1235.33	20.59	84	

*P* value is based on 1000 permutations; df = degree of freedom

**Fig 2:**
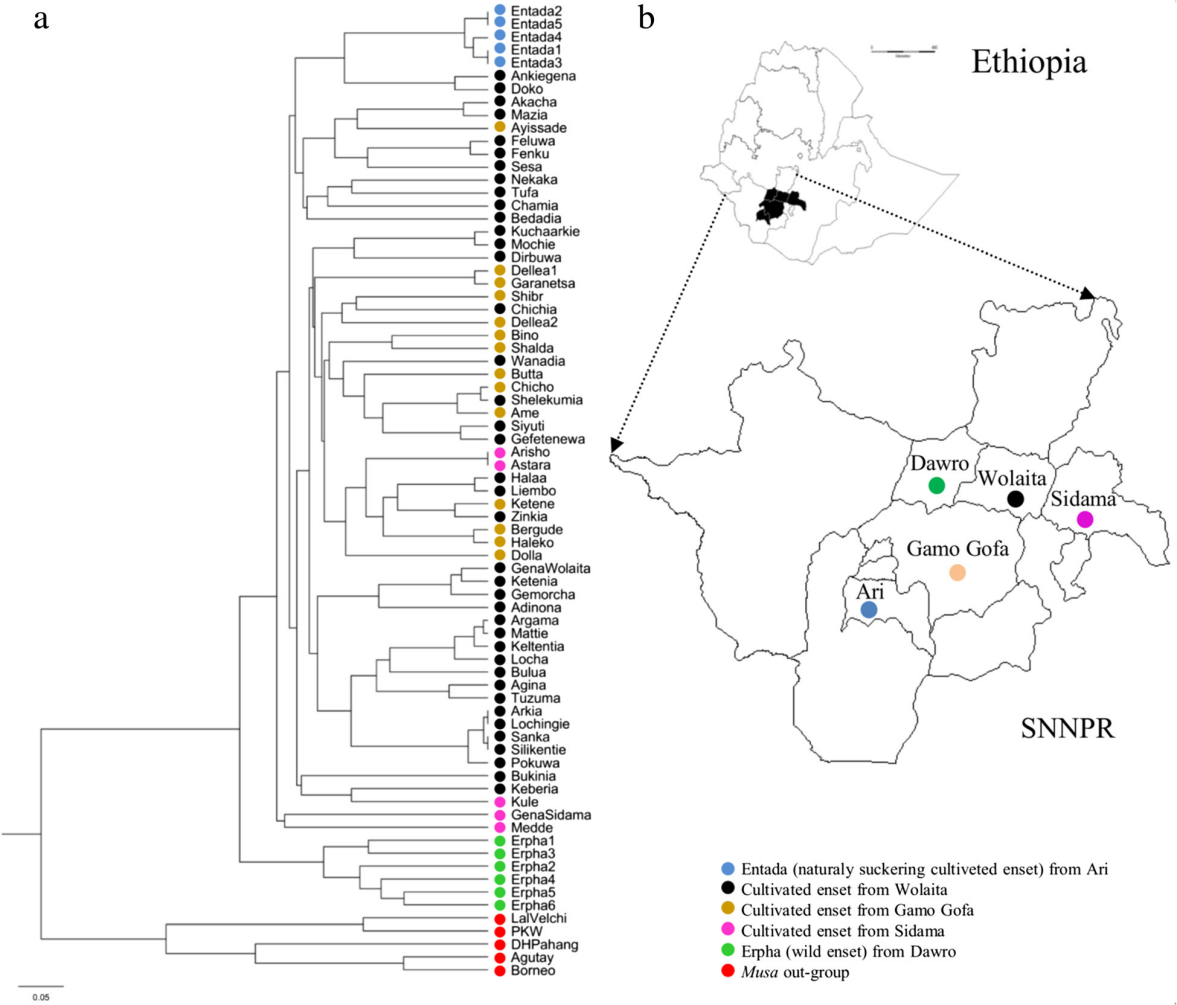
Genetic relationship and its pattern across sampling regions. **a**: UPGMA phylogenetic tree of individuals based on 34 polymorphic markers, **b**: Geographical location of sampling distribution. The colors of the dots in the tree correspond to the sampling location Southern Nations Nationalities and People’s Region, in southern Ethiopia

**Fig 3:**
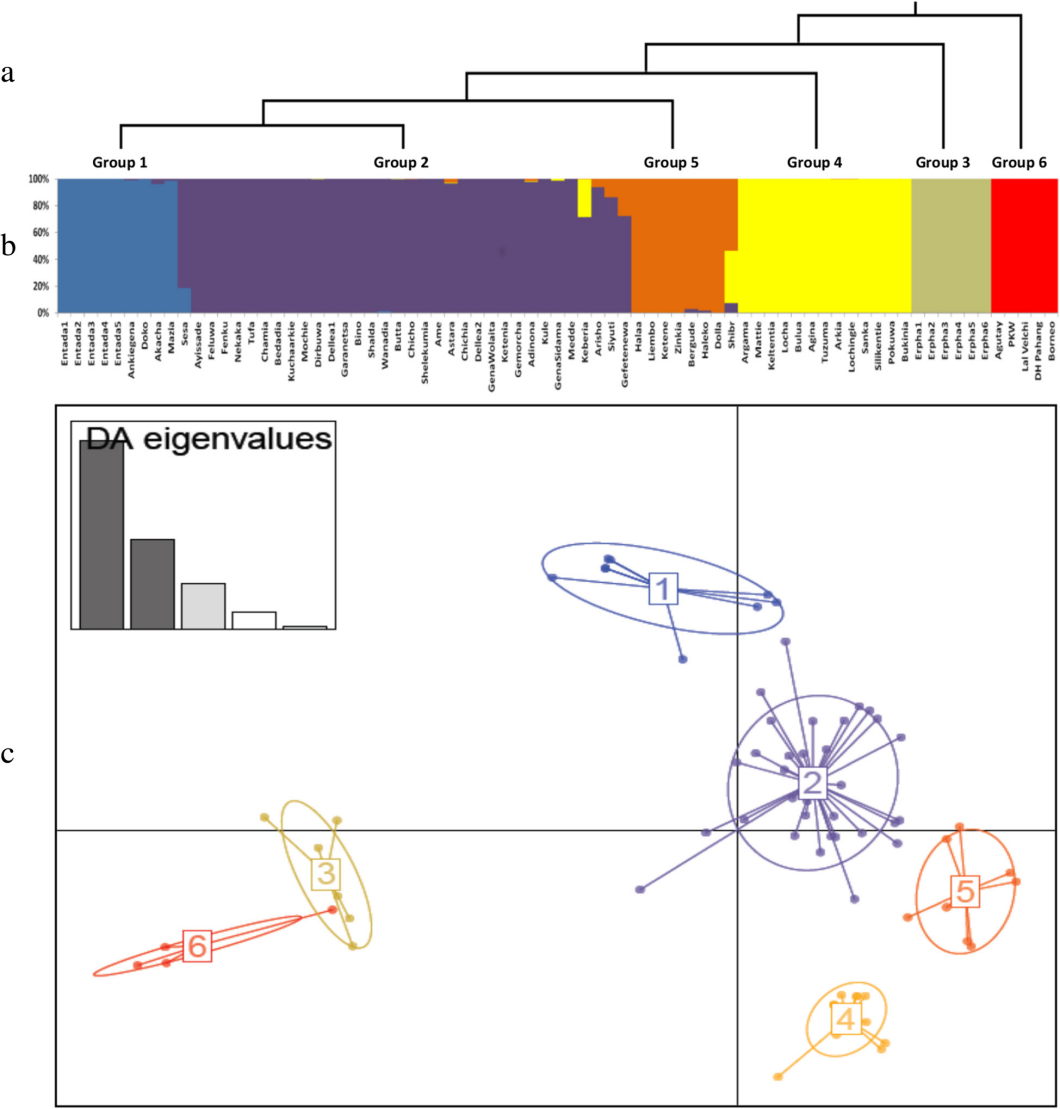
Population structure based on 34 polymorphic SSR markers. **a**: phylogenetic tree of 5 enset groups and out-grouping *Musa* accessions inferred from DAPC, **b**: The estimated group structure with individual group membership values, **c**: DAPC scatter plot for 70 enset and 5 *Musa* accessions using the first two PCs. The inset indicates the number of PCs retained to describe the relationship between the clusters. The DAPC population numbers in each of the clusters correspond to group numbers of the phylogenetic tree

UPGMA cluster (Fig. 2) and DAPC (Fig. 3) analyses showed interesting and consistent patterns of genetic relationship and differentiation among the assessed cultivated enset groups from the four growing regions and the wild (*Erpha*) group from Dawro. In UPGMA, clustering using genetic distance-based analysis by calculating Nei’s coefficient, all enset accessions clustered distinctly away from the five *Musa* accessions included as an out-group. Within enset accessions, genetic clustering reflected the domestication status of enset, as illustrated by the distinct grouping of wild enset (*Erpha*) from cultivated landraces. Cultivated enset landraces further showed some distinction between spontaneously suckering *Entada* and induced suckering landraces, but no distinction based on cultivation regions.

Most cultivated landraces grouped sporadically without a specific cluster pattern associated with the growing regions, thus reaffirming the AMOVA results, which showed a small genetic variation between regions. Overall, the average distance based on the 34 markers among the accessions was 0.42 and ranged from 0.00 to 0.70, indicating that there was a moderate to high amount of genetic variation. Some landraces did not differ in their SSR profile for the tested markers, including *Astara*/*Arisho*, *Arkia*/*Lochingia*, *Sanka*/*Silkantia* (Fig. 2a). On the other hand, two landraces identically named as *Gena* in Sidama and Wolaita growing zones showed different SSR profiles, with a genetic distance of 0.60, thus indicating a case of homonymy.

As expected, the genetic distance among the five *Entada* individuals was very narrow, ranging from 0.00 (*Entada1*/*Entada3* and *Entada2*/*Entada5*) to 0.08 (*Entada2*/*Entada5*). Based on the DAPC clustering analysis, six clusters (K = 6) were identified as being optimal to describe the full set of data (Additional file 5). One of the clusters only included the *Musa* spp. accessions, another one contained only wild enset individuals. All cultivated landraces derived from the four growing regions were included in the remaining four clusters, irrespectively of the geographic region from where they were originally collected. More than half (34/64) of the enset landraces were grouped together into one cluster, including five landraces from Sidama, 11 from Gamo Gofa, and 18 from Wolaita.

### SSR marker cross-genera transferability

**Fig 4:**
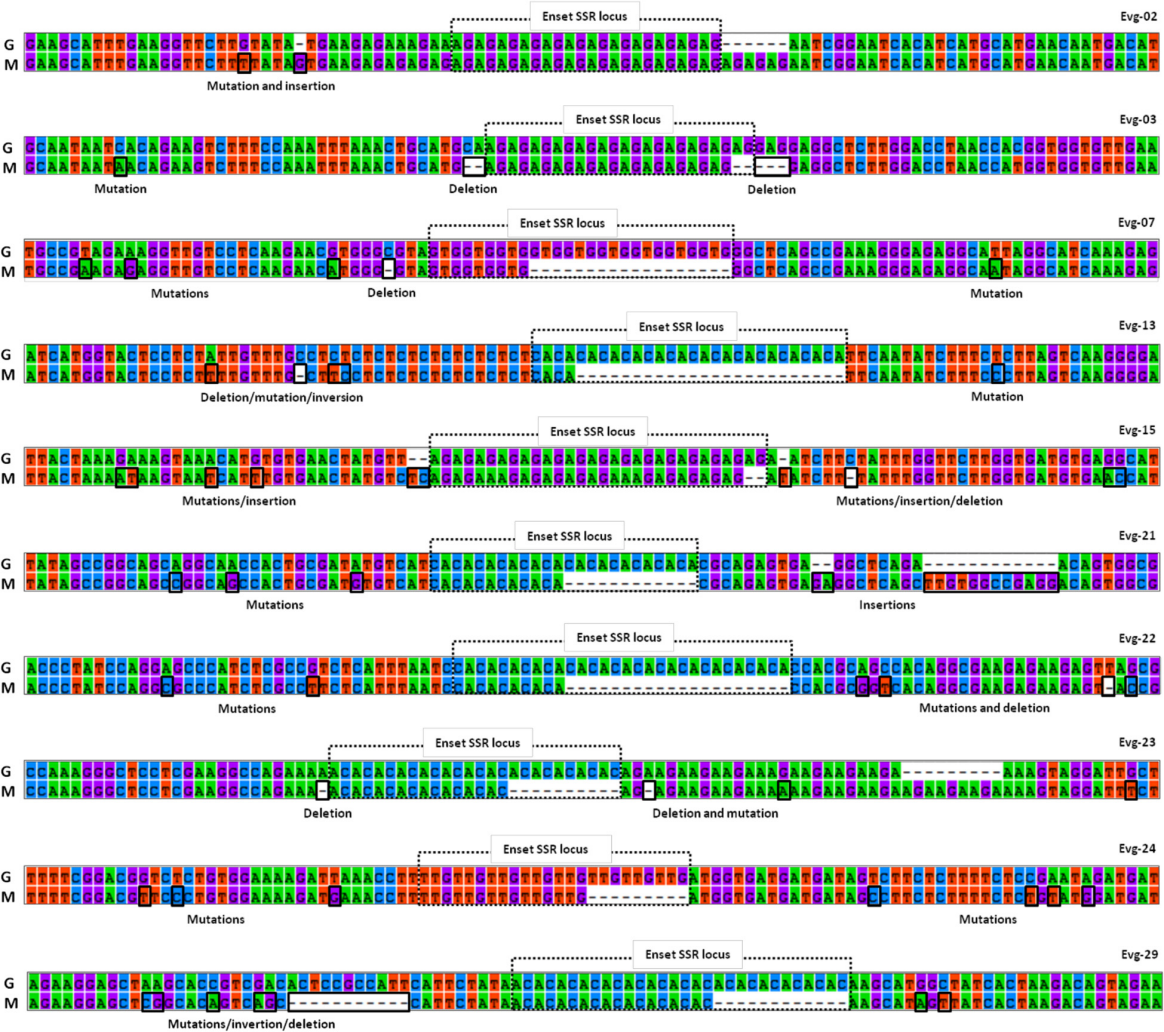
Alignment and comparison of SSR containing homologous sequences between *E. ventricosum* landrace *Gena* (G) and *M. acuminata* ssp. malaccensis (M). Rectangular boxes indicate the occurrence of a variable number of repeat motifs between the two species along with multiple point mutations and INDELs both in SSR repeat block and flanking regions

To determine the usefulness of the developed SSR markers beyond *E. ventricosum*, we tested the 34 enset SSR markers on 18 *Musa* accessions representing five species from two different taxonomic sections. Fourteen of the 34 enset SSR markers amplified PCR products in *Musa* accessions. To locate and verify the amplified SSR loci in *Musa*, a computational search over the genome sequence of *M. acuminata* [GenBank: CAIC01] was performed in the NCBI BLASTN, using the enset sequences on which primer pairs were designed. Subsequent alignment of the resulting hit in the program MEGA 5.1 showed a high degree of sequence homology and the presence of SSR motifs for 10 of the SSR markers. For these 10 verified cross-genus transferable SSR markers, pair-wise aligned orthologous sequences of *E. ventricosum* and *M. acuminata* showed a few variations, such as a number of repeated motifs, base substitution/transitions and/or INDELs (Fig. 4). For the remaining four of 14 cross-amplifying markers, SSR motifs were either completely absent or showed a high degree of mutation and/or INDELs in the orthologous sequences of *M. acuminata* (data not shown). Nine of the verified and consistently cross-amplified enset SSRs showed a high level of polymorphism across the 18 *Musa* accessions, identifying 65 alleles, with an average of 7.22 alleles and PIC values ranging from 0.63 (Evg-13 and Evg-22) to 0.86 (Evg-03), with an average of 0.75. The amplification pattern of enset SSRs on the five *Musa* species is provided in the Additional file 6*.* In a further analysis performed to verify the discriminatory capacity of the cross-transferable markers using Nei’s genetic distance, the markers were able to recapitulate the known phylogenetic relationship among the tested *Musa* accessions (Additional file 7).

## Discussion

### Development of enset SSR markers

The first set of enset SSR markers was produced using *454* pyrosequencing of microsatellite enriched genomic libraries. Enrichment procedure is reported to increase the likelihood of detecting microsatellites, especially in species with unstudied microsatellite composition, as is the case of enset [24, 38]. The enset libraries were enriched for AC/CA and AG/GA SSR motifs, as previous studies have reported the prevalence of dinucleotide repeats with AG/CT motifs and the rarity of AT/CG motifs in plant genomes, *Musa* included [39, 40]. Recently, other studies have also applied SSR enriched genomic DNA pyrosequencing to develop SSR markers for genetically understudied non-model crop species, such as grass pea (*Lathyrus sativus* L.) [41] and Andean bean (*Pachyrhizus ahipa* (Wedd.) Parodi) [42]. The success of this approach in enset is demonstrated by the high number (840) of SSR-containing sequences identified from less than 10,000 generated reads. From those 840 reads, we were able to design 217 hypervariable SSRs (Table 1, Fig. 1) [25]. Given the fact that we selected only a few classes of SSRs (di-, tri- and tetra- nucleotide SSRs with a repeat motif of > 20 bp) and we used highly stringent procedures for their validation (see Methods), our sequence data, publicly available in Sequence Read Archive [GenBank: SRR974726], could be used to develop additional SSR markers for enset or other type of genetic markers such as SNPs (Single Nucleotide Polymorphism) in combination with other available enset genome sequences.

Among the identified SSRs, (AG/GA)_n_ and (AAG/AGA/GAA) were the dominant di- and tri-nucleotide motifs respectively, whereas (CG/GC)n, (CCG/CGG)n were rarely detected (Fig. 1). This result is in agreement with SSR frequency and distribution observed in several other plant species [39–41]. However, the limited genomic coverage and the enrichment applied in the present study prevent any generalization regarding the genome wide SSR composition of enset. Indeed, genomic composition and abundance of SSR motifs differ depending on the many variables involved in a given study, including the depth of sequence employed*,* the type of probes used in the SSR enrichment, and the software criteria used for mining SSRs [38, 43].

Adopting a combined approach based on *in silico* PCR [44–46] using the publicly available genome sequences of enset and *in vitro* PCR amplification, a total of 59 primer pairs able to uncover polymorphism were validated.

The *in silico* approach enabled us to quickly test all the 217 designed primer pairs and at virtually no cost. However, a smaller proportion (24 %, 52 out of 217 tested primers) of the primers were validated in the *in silico* than in the *in vitro* PCR (71 %, 34 out of 48 tested primers). This discrepancy might be related, for example, to the template sequences that were used in the *in silico* strategy. The less fragmented enset genome sequences that are available in the GenBank database and used as templates are 1/3 [GenBank: AMZH01] and 2/3 [GenBank: JTFG01] of the estimated complete enset genome size (547 megabases), which would potentially result in missing loci by primer pairs [29]. Other factors that might have contributed to this difference could be the genetic distance and associated inefficiency of primer pair annealing on the template sequence. In fact, more primer pairs produced an amplicon in a cultivated *Bedadit* template sequence than in the uncultivated sequence. The larger sample size (*n* = 10) used to validate the primers in the *in vitro* approach compared to the two PCR primer template sequences used in the *in silico* strategy might also have favored the number of validated primers in the *in vitro* approach. However, despite the difference in the number of validated primer pairs, the experimental *in vitro* PCR results were largely consistent and complementary with those of the *in silico* PCR.

### Genetic diversity among enset accessions

Thirty-four experimentally validated enset SSR markers were used for the first time to assess intra-specific enset genetic diversity in 60 cultivated landraces and six wild individuals.

The collection in our study represented over 20 % of the landraces in the long-term enset germplasm maintained at AARC (Areka, Ethiopia). The 34 enset SSR markers detected a total of 202 alleles in the assessed collection (Table 2), and a large proportion of them (76 %, 26 out of 34 SSRs) also exhibited PIC values of > 0.5, making them a highly informative marker set for population genetic studies. The extent of allele numbers is particularly high, compared to only 61 alleles identified in 220 accessions using 11 *Musa* markers [47]. Similarly, the level of genetic diversity, as quantified by the mean expected heterozygosity, was slightly higher for SSR markers specifically developed in enset (*He* = 0.59; Table 1) than for the cross-transferred *Musa* SSRs (*He* = 0.55) [47].

The level of genetic diversity estimated using SSR markers is higher than previous reports for other DNA markers [13–15]. This is expected as SSR are more variable markers than RAPD, AFLP and ISSR [17]. However, the difference in number and type of the accessions and DNA markers, makes a direct comparison between these studies difficult to draw general conclusions. In our study the observed mean heterozygosity was 0.55 (Table 2), which is consistent with the out-crossing nature of enset. It is interesting to note that the highest level of heterozygosity was observed in the *Erpha* samples, corresponding to wild enset accessions, which are sexually multiplied by seeds (Table 4). The generally high heterozygosity in enset is typical as in other naturally out-crossing, perennial species that are highly selected for cultivation and then clonally propagated [48, 49].

The enset markers revealed a 29 % cross-genus amplification rate (10 out of 34 tested). Nine of these were polymorphic in the 18 *Musa* accessions analyzed. Cross-genera amplicons for enset SSRs were verified by sequence homology and the presence of an SSR motif region in the *M. acuminata* genome sequence [30]. Variations in the numbers of repeat motifs, base substitution/transitions, INDELs were observed both in flanking sequences and motif regions. Such variations have been previously reported for cross-genus amplifying *Musa* SSRs when tested on the genus *Ensete* including *E. glaucum* (Roxb.) Cheesman [50] and *E. ventricosum* (Welw*.*) Cheesman [47]. The availability of cross-genera transferable SSRs between *Ensete* and *Musa* is useful for intra- and inter-genera evolutionary studies and could contribute to refine the taxonomic and phylogenetic relationship in the Musaceae family.

The study of the population structure and genetic relationships among wild enset and cultivated landraces from different ethno-linguistic communities or regions provide useful information on the putative domestication events, evolutionary relationships, or gene flow events in enset. The UPGMA tree (Fig. 2a) and the DAPC scatter plot (Fig. 3) both revealed a high level of differentiation between wild and cultivated enset. Other studies have also reported a genetic divergence between cultivated and wild enset [22]. Our results confirm the acknowledged hypothesis of a highly restricted landrace-wild gene flow, due to both the natural distribution of wild enset, as well as the farming and management practices of cultivated landraces [22]. It should be noted that wild enset mainly occurs in forests, river banks, swamps and ritual sites, mostly a long way from the home gardens harbouring cultivated landraces [9, 51]. In addition, farmers’ practices of vegetatively propagating enset and harvesting the crop before it flowers, further restrict any cross-fertilization with sexually reproducing wild enset [22].

The cultivated enset landraces showed a low differentiation according to the geographic region of their original collection, as consistently revealed by the AMOVA results (Table 5), UPGMA tree (Fig. 2a) and DAPC scatter plot (Fig. 3). AMOVA revealed that the proportion of variance within the growing geographic regions contributed by 84 % to the total genetic variation. These results imply that genetic variation in enset landraces is less affected by the region of origin, which is in agreement with previous reports [13, 15, 20]. For instance, AFLP analysis of 146 enset landraces from five growing regions showed a limited proportion of variation among growing regions (4.8 %), but a considerable variation (95.2 %) within regions [15]. Similarly, for enset accessions collected from eight zones, *Musa* SSRs attributed low and high proportions of genetic variation to among groups and within groups comparisons, respectively [47].

The observed low divergence of enset landraces from different growing regions could be partly explained by gene flow, the common origin of the populations, or the extensive exchange of enset planting materials, which exists among different enset growing communities [9, 51, 52]. The domestication of enset, as in many other clonally propagated crops, rarely leads to speciation [53]. The postulated process of domestication in enset involves the selection of individuals from wild populations that maintain sexual reproductive systems with frequently flowering plants on the basis of desirable morpho-agronomic characters. Once identified and selected, the wild individuals are brought to home gardens, named and added to cultivated landraces and maintained through vegetative propagation. Any further new domesticates are given the same name if similar to the existing landrace, or different names if they differ in morpho-agronomic characteristics from existing landraces. The new individuals could therefore become new landraces or additions to known landraces, and be distributed though ‘seed’ exchange networks to other communities [51].

The results support this hypothesized domestication and gene flow in enset, and imply that the selection of enset landraces for breeding and improvement programs should be based on actual genetic distances, and not based on growing regions.

The existence of synonyms, homonyms and associated mislabeling is an important challenge for the germplasm conservation of crop species. This is particularly important for regions with rich ethno-linguistic diversity, where a cultivated plant is extensively shared among communities with its local name either retained or changed [54]. In the enset farming systems of southern Ethiopia, many ethno-linguistic communities cultivating enset give vernacular names to landraces according to their own language, and exchange planting materials within and beyond their own communities, irrespective of geographical distances [9, 52]. In fact, there are reports on homonyms, synonym duplicates and their associated challenges in the germplasm management of enset genetic resources [12]. For instance, in the AFLP based analysis of 140 landraces collected from farmers’ fields in 5 regions, 21 duplicates involving 58 landraces were encountered [15]. In the present study, two landraces identically named as *Gena* in Sidama and Wolaita that revealed a genetic distance of 0.6 were identified as possible homonyms. Conventional morphological and agronomic evaluations supported the differences observed between *Gena* from Sidma and Wolaita [11].

On the other hand, three pairs of landraces (*Arkia*/*Lochingia*, *Sanka*/*Silkantia* and *Astara*/*Arisho*) showed no difference in the SSR profile. However, the former two pairs were reported to show clear morpho-agronomic variability under the same environmental conditions [9]. This contradiction might be related to the limitation of the morphological classification of germplasm in which the characteristics are easily affected by environmental conditions. However, differences in microsatellite polymorphisms may not necessarily correspond to variations in morphological or agronomic traits as reported in *Musa* spp. [55]. Thus, interdisciplinary approaches are needed in order to integrate the conventional evaluation of morphological and physiological traits or other nutrient composition/organoleptic characteristics of enset landraces, in addition to neutral DNA markers. Such approaches could then be used for identifying duplicates and useful genotypes, and for defining core germplasm sets for enset.

The co-dominant markers that were generated in the present study are a promising resource, not only for the genomic fingerprinting of enset landraces, but also for identifying and developing reliable germplasm sources for breeding programs. More SSR markers need to be developed and mapped for marker-assisted selection strategies in order to accelerate the improvement of the enset crop.

## Conclusions

The present study contributes fundamental information for the implementation of appropriate conservation plans and breeding programs for enset genetic resources. The first set of SSR markers was developed from the genomic sequences of *E. ventricosum* and applied in genetic diversity and structure analyses in one of the most important enset germplasm collections in Ethiopia. Our enset SSR markers are cross-genus transferable to *Musa* spp. and can be useful for genetic studies in the Musaceae family.

The molecular data indicated that the wild and cultivated enset landraces are very diverse. The patterns of genetic variability in cultivated enset landraces are not associated with cultivation regions, which is in agreement with the postulated enset domestication and extensive enset seed-sucker exchange systems in southern Ethiopia. The information is a timely contribution, considering enset’s high food security value, greatly confined endemism and current challenges in enset biodiversity management and conservation.

## Availability of supporting data

The sequence data set obtained by pyrosequencing of *E. ventricosum* landrace *Gena* genomic libraries and supporting the results of this article is available in the GenBank SRA repository, [GenBank: SRR974726] http://www.ncbi.nlm.nih.gov/sra/?term=SRR974726.

The data set of 67 SSR markers developed from the genomic sequences of *E. ventricosum* is available in the GenBank Probe repository, from [GenBank: Pr032360175] http://www.ncbi.nlm.nih.gov/probe/pr032360175 to [GenBank: Pr032360241] http://www.ncbi.nlm.nih.gov/probe/pr032360241.

The phylogenetic data are available in TreeBASE: http://purl.org/phylo/treebase/phylows/study/TB2:S17807.

## Additional files

Additional file 1:
**Descriptions of the 70 enset (** ***Ensete ventricosum*** **(Welw.) Cheesman) plant materials used for genetic analysis.**
Additional file 2:
**Descriptions of the 18** ***Musa*** **accessions used for marker** **cross-transferability** **evaluation and genetic analysis.**
Additional file 3:
**Characteristics of the 217 designed primer pairs from sequence reads of enset (** ***Ensete ventricosum*** **(Welw.) Cheesman) landrace** ***Gena***. Additional file 4:
**Features of newly developed enset SSR markers.** The data provided represent details of the new SSR markers e.g. marker name, primer sequence, primer annealing temperature, indication of repeat type, expected product size and primer validation method. Additional file 5:
**Discriminant Analysis of Principal Components (DAPC). A:** inferences of the number of clusters (K -groups) in the DAPC performed on the dataset of 70 enset and 5 *Musa* out-group accession; K value of 6 (at the lowest BIC value) represents the optimal clusters for summarizing the data. **B:** Optimization α-score graph for retained PCs. **C:** Group membership and size graph for the inferred number of K clusters. Additional file 6:
**Characteristics and amplification pattern of** **cross-transferable** **enset** (***Ensete ventricosum*** **(Welw.) Cheesman) SSR markers in** ***Musa*** **spp.**
Additional file 7:
**Phylogenic relationship among 18** ***Musa*** **accessions based on 9 polymorphic SSR markers from** ***E. ventricosum***. The colored dots denote correspondence of individual accession to their respective species or cultivar groups.

## Abbreviations

AMOVA: Analysis of molecular variance
AARC: Areka Agricultural Research Centre
DAPC: Discriminant Analysis of Principal Components
Evg: *Ensete ventricosum* landrace *Gena*
HwU: Hawassa University
ITC: International Transit Center for *Musa* collection
SNNPR: Southern Nations, Nationalities and peoples’ Region
UPGMA: Unweighted Pair-Group Method with Arithmetic mean
