# Supplementary material for: Development of SSR markers and genetic diversity analysis in enset (Ensete ventricosum (Welw.) Cheesman), an orphan food security crop from Southern Ethiopia
Source: BMC Genet. 2015 Aug 5;16:98. doi: 10.1186/s12863-015-0250-8 (PMC4524394; doi:10.1186/s12863-015-0250-8)
Supplement: Additional file 5: — Discriminant Analysis of Principal Components (DAPC). A: inferences of the number of clusters (K -groups) in the DAPC performed on the dataset of 70 enset and 5 Musa out-group accession; K value of 6 (at the lowest BIC value) represents the optimal clusters for summarizing the data. B: Optimization α-score graph for retained PCs. C: Group membership and size graph for the inferred number of K clusters. [file 12863_2015_250_MOESM5_ESM.pdf]

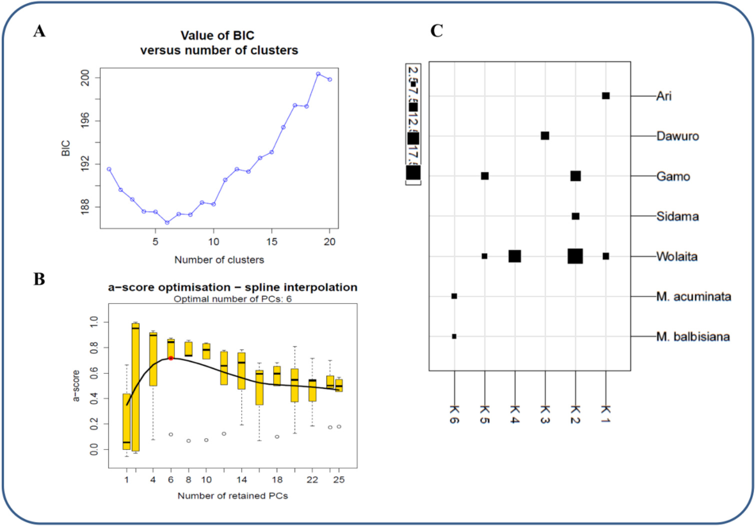
A
Value of BIC
versus number of clusters
BIC
200
196
192
188
5
10
15
20
Number of clusters
B
a−score optimisation − spline interpolation
Optimal number of PCs: 6
a−score
1.0
0.8
0.6
0.4
0.2
0.0
1
4
6
8
10
14
18
22
25
Number of retained PCs
C
Ari
Dawuro
Gamo
Sidama
Wolaita
M. acuminata
M. balbisiana
K 6
K 5
K 4
K 3
K 2
K 1
