# Supplementary figures and images for: Development of SSR markers and genetic diversity analysis in enset (Ensete ventricosum (Welw.) Cheesman), an orphan food security crop from Southern Ethiopia
Source: BMC Genet. 2015 Aug 5;16:98. doi: 10.1186/s12863-015-0250-8 (PMC4524394; doi:10.1186/s12863-015-0250-8)

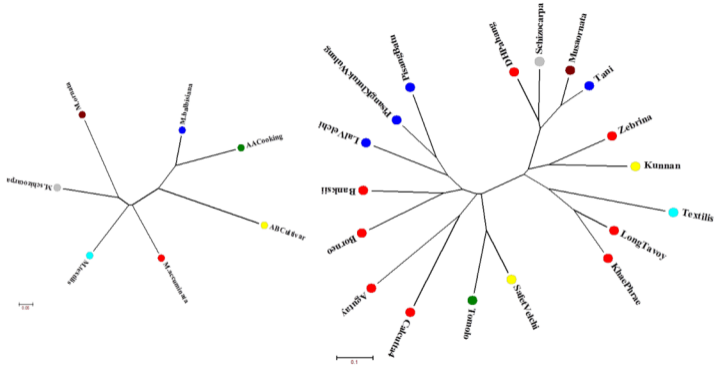

Supplement: Additional file 7: — Phylogenic relationship among 18 Musa accessions based on 9 polymorphic SSR markers from E. ventricosum. The colored dots denote correspondence of individual accession to their respective species or cultivar groups. [file 12863_2015_250_MOESM7_ESM.pdf]
